# Safety and Effectiveness of Triple-Antenna Hepatic Microwave Ablation

**DOI:** 10.3390/bioengineering11111133

**Published:** 2024-11-11

**Authors:** Nikola Bošković, Srdjan Nikolić, Branislav Radjenović, Marija Radmilović-Radjenović

**Affiliations:** 1 Institute of Physics, University of Belgrade, Pregrevica 118, 11080 Belgrade, Serbia; nikolab@ipb.ac.rs (N.B.); bradjeno@ipb.ac.rs (B.R.); 2 Department of Surgery, Institute of Oncology and Radiology of Serbia, Pasterova 14, 11000 Belgrade, Serbia; onkosurge1@yahoo.com; 3 Faculty of Medicine, University of Belgrade, Dr Subotica 8, 11000 Belgrade, Serbia

**Keywords:** computational physics, microwave ablation, necrotic tissue, open-source software

## Abstract

Microwave ablation is becoming a standard procedure for treating tumors based on heat generation, causing an elevation in the tissue temperature level from 50 to 60 °C, causing tissue death. Microwave ablation is associated with uniform cell killing within ablation zones, multiple-antenna capability, low complication rates, and long-term survival. Several reports have demonstrated that multiple-antenna microwave ablation is a promising strategy for safely, rapidly, and effectively treating large tumors. The key advantage of multi-antenna tumor microwave ablation is the creation of a large, well-defined ablation zone without excessively long treatment times or high power that can damage healthy tissue. The strategic positioning of multiple probes provides a fully ablated volume, even in regions where individual probe damage is incomplete. Accurate modeling of the complex thermal and electromagnetic behaviors of tissue is critical for optimizing microwave ablation because material parameters and tissue responses can change significantly during the procedure. In the case of multi-antenna microwave ablation, the calculation complexity increases significantly, requiring significant computational resources and time. This study aimed to evaluate the efficacy and safety of liver percutaneous microwave ablation using the simultaneous activation of three antennas for the treatment of lesions larger than 3 cm. Based on the known results from a single-probe setup, researchers can estimate and evaluate various spatial configurations of the three-probe array to identify the optimal arrangement. Due to the synergistic effects of the combined radiation from the three antennas, the resulting ablation zone can be significantly larger, leading to better outcomes in terms of treatment time and effectiveness. The obtained results revealed that volumetric damage and the amount of damaged healthy tissue are smaller for a three-antenna configuration than for microwave ablation using a single-antenna and two-antenna configurations.

## 1. Introduction

Microwave ablation (MWA) is a minimally invasive thermal ablation modality for treating various types of tumors [1,2,3,4,5]. MWA is based on tumor cell destruction via hyperthermia caused by microwave radiation emitted by a small probe inserted into the tumor [6]. The success of MWA relies on carefully balancing various technical parameters to achieve efficient tumor destruction while sparing the healthy surrounding tissue. The key parameters affecting tissue damage are the input power, treatment duration, geometry, position of the probe, and radiation propagation characteristics of the antenna [7,8,9].

Single-antenna and multi-antenna MWA’s showed similar effectiveness for local treatment of liver tumors. Nevertheless, synchronous multi-antenna MWA exhibited better local tumor progression-free survival as compared to other MWA approaches, particularly for larger liver tumors [10,11,12]. For large tumors, the use of a single probe is not recommended, requiring extreme power and/or treatment duration, thus causing excessive damage to healthy tissues [13]. There are two main approaches for the treatment of large tumors [14,15]. The simplest method is to use a single probe at multiple points, creating overlapping smaller ablation zones to form one large ablation zone that lasts longer than standard MWA. The second approach uses multiple probes simultaneously, creating a larger, more uniform, and symmetrical ablation zone compared to the sequential single-probe approach. The procedure duration was the same as single-probe MWA, but the overlapping radiation zones achieved with multiple probes result in a much higher density of the electromagnetic field [16].

The choice of the radiating antenna is critical for providing efficient radiation in high-density tissue media [17,18]. Coaxial slot antennas are commonly used, with multi-slot antennas typically exhibiting better radiation patterns to create more predictable spherical ablation zones [19,20]. In a real-time multi-probe setup, the probes are generally placed in one of two configurations in a straight line with identical probe separation, for elongated tumors, or in an equilateral geometric shape like a triangle or square, for large irregular tumors [21]. The focus is on understanding the development of the ablation zone in a multi-probe setup.

Computer modeling plays a critical role in improving the precision and effectiveness of MWA by providing a deeper understanding of thermal spread, which is crucial for successful clinical outcomes [22,23,24,25]. It was demonstrated that two-dimensional (2D) calculations are not sufficient to estimate the optimal input power and ablation time [20,25]. Thus, it is necessary to perform three-dimensional (3D) simulations, taking into account not only the tumor geometry and the antenna design but also properties of healthy and tumoral tissues [20,25].

In this study, we used the SimSurgery simulation environment based on open-source scientific computing libraries to determine the optimal parameters required for efficient microwave ablation [26,27]. The finite element method (FEM) was used to calculate the processes during MWA. The FEM involves segmenting the entire domain into small finite elements, and the number and quality of these elements directly affect the duration and accuracy of the FEM numerical method [28,29,30]. Calculations were performed for the triple-antenna MWA of a real liver tumor (from the database [31]). For complex simulations involving a probe, real-life tumor, and part of the liver, the required computational resources can be high. With a multi-probe setup, there are many possible combinations, and as the number of probes increases, the number of finite elements increases significantly, making the full FEM simulation of each setup unacceptably long. The critical challenge seems to be the high computational cost and time required to perform detailed FEM simulations for all the possible multi-probe configurations, given the complexity of the real-life tumor and the geometry involved.

## 2. Methodology

### 2.1. Theoretical Background

The material parameters of the tissue dictate the propagation of electromagnetic waves. Liver tissue has a high water content of approximately 70%. As the heating water content decreased, the material parameters change drastically [32]. Blood perfusion is another major factor affecting tissue heating. The blood flow behaved as a coolant, significantly affecting the temperature distribution. There are differences between the material parameters of healthy tissue and tumor, and these parameters change drastically with temperature [33,34]. Instantaneous cell death occurs at temperatures greater than 60 °C, and blood flow stops due to coagulation. Exposure to temperatures above 50 °C for a moderate duration can cause cell death, whereas prolonged exposure to temperatures above 42 °C can cause permanent tissue damage. Determining tissue damage as a function of temperature is a complex task. The calculation of the MWA process can be divided into three stages: calculation of the propagating electromagnetic field inside the tissue, calculation of the temperature, and estimation of thermal damage [35].

The electric field distribution inside the domain can be determined by solving Maxwell’s equations [35]:

(1) $$\nabla^{2}\vec{E}-\mu_{r}k_{0}^{2}\left(\varepsilon_{r}-\frac{j\sigma }{\omega \varepsilon_{0}}\right)\vec{E}=0,$$

where $\mu_{r}$ is the relative permeability, $\vec{E}$ is the electric field vector, σ is the electric conductivity, $\varepsilon_{0}$ is the permittivity of vacuum, ω=2πf is the angular frequency, and $k_{0}$ is the vacuum propagation constant. 

Pennes’ bio-heat equation describes temperature changes due to microwave radiation [35,36]:

(2) $$\rho \left(c-\frac{\alpha }{\rho }\frac{\partial W}{\partial T}\right)\frac{\partial T}{\partial t}=\nabla \cdot \left(k\nabla T\right)+\rho_{b}\omega_{b}c_{b}\left(T_{b}-T\right)+Q_{ext}+Q_{met},$$

where α, W, ρ, c, T, *t*, and *k* are the water latent heat constant, change in water content with temperature, density, specific heat capacity, temperature of the tissue, time, and thermal conductivity of the tissue, respectively. $\rho_{b}$, $\omega_{b}$, and $c_{b}$ represent the density, perfusion rate, and specific heat capacity of the blood, respectively. $T_{b}=$ 37 °C is the arterial blood temperature, and $Q_{met}$ is the metabolic heat, which is much lower than $Q_{ext}$, which represents the external heat source; hence, it can be neglected.

The specific absorption rate (SAR) is defined as the energy of the EM field divided by mass density, representing a measure of the radiation absorbed by the tissue [35]:

(3) $$SAR=\frac{\sigma }{2\rho }{\left|\vec{E}\right|}^{2}.$$

where $\vec{E}$ is the electric field vector, *σ* is electric conductivity, and *ρ* is the density of the observed domain. The SAR can identify the tissue that is most affected by radiation and how the electric field propagation interacts with the tissue. 

Tissue damage accumulates over time and is a function of both temperature and duration of exposure and can be estimated according to the Arrhenius form [37]:

(4) $$\frac{\partial \Omega }{\partial t}=Ae^{\left(-\frac{\Delta E}{RT}\right)},$$

where *R* and *T* are the gas constant and temperature, respectively. *A* is the frequency factor, while Δ*E* is the activation energy of the irreversible damage reaction. 

Two expressions were used to estimate damage to healthy tissue. The volumetric damage (*VD*) was calculated as the ratio of the volume of the damaged healthy tissue (*V_DAMAGED_*) and tumor tissue (*V_TUMOR_*) [38,39]:

(5) $$VD=\frac{V_{DAMAGED}}{V_{TUMOR}}\cdot 100\%.$$

The amount of damaged healthy tissue (*DT*) was calculated as the ratio of the volume of the damaged healthy tissue to the total volume of the liver (*V_LIVER_*) without tumor tissue (*V_TUMOR_*) [38,39]:

(6) $$DT=\frac{V_{DAMAGED}}{V_{LIVER}-V_{TUMOR}}\cdot 100\%.$$

### 2.2. Simulation Conditions

**Figure 1:**
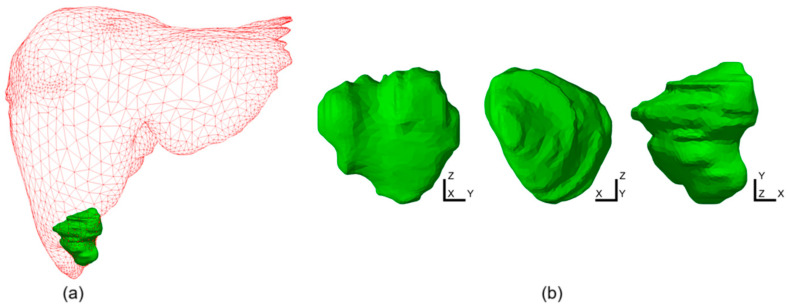
(**a**) Liver (red triangular surface) and tumor (solid green surface). (**b**) Tumor STL (stereolithography) representation obtained from the patient in different planes. The dimensions of the tumor are 31.6 mm × 35.4 mm × 33.9 mm [31].

**Figure 2:**
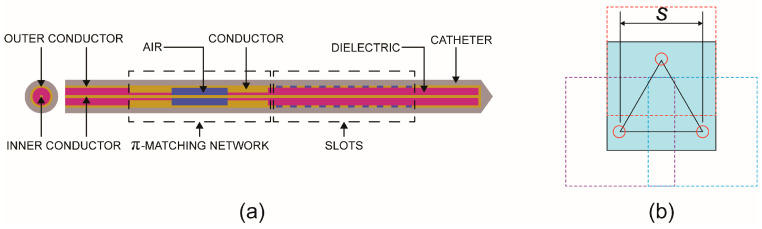
(**a**) Schematic views of the 10-slot microwave antenna; (**b**) The three-antenna configuration (blue surface) represents the cross-section of the domain in the *xy*-plane, red circles represent probes, and dashed lines illustrate domains around the probes.

In this study, we compared the efficacy of single-probe vs. multi-probe MWA of a large liver tumor in a female patient born in 1987 (from the IRCADb-01 liver tumor database [31]). The tumor was irregularly shaped and large (31.6 mm × 35.4 mm × 33.9 mm) with the position and shape shown in Figure 1. The probes were oriented along the z-axis. With a fixed duration of MWA of 600 s with a single probe, the only modality that changed the ablation size was the input power change. We performed a simulation for a three 10-slot antenna configuration placed inside the probe as the source of radiation. The 10-slot antenna (Figure 2a) for microwave ablation represents an advancement in minimally invasive cancer treatment, providing precise and effective tissue destruction with the potential for improved patient outcomes [35]. The multi-slot antenna contains several periodic elements equal to a linear uniform antenna array. Each periodic element includes a slot with a width of 0.6 mm, and there is a conductor spacing of 0.8 mm between neighboring slots. The antennas operate at a frequency of 2.45 GHz, belonging to the ISM bands (Industrial, Scientific, and Medical bands), which is the most commonly used frequency in MWA procedures. Power generators operating at 2.45 GHz are easily available, small antennas with a few millimeters in diameter can be made, and electromagnetic waves at 2.45 GHz can penetrate deep into tissue [40].

If we calculate a single probe in the entire domain, multiply this domain, and spatially translate all finite elements around the probe to the positions of the probes in the multi-probe setup, we can obtain a large virtual computational domain consisting of *n* subdomains and *n* probes. We can then sum all fractions of tissue damage in the *n* subdomains and create a large computational domain with the solid first estimation of ablation in a specific multi-probe setup. This of course would never be identical to a complete FEM simulation with a multi-probe setup due to the exiting mutual coupling of the probes. Calculations of this type can be performed almost instantaneously for any number of spatial combinations as they do not require costly FEM simulation but simple numerical post-processing of the existing results. Hence, we can identify the best possible combination for the full FEM simulation. 

The computational domain size was 60 mm × 60 mm × 90 mm and has the shape of a hexahedron. From the simulation of necrosis with a single probe, we obtained the distribution of the fraction of necrotic tissue in the simulation domain (blue square in Figure 2b). Each finite element in the domain is assigned a corresponding fraction of necrotic tissue. If we position the center of the system at the position of (0, 0) in the *xy*-plane, the centers of the probes in the equilateral triangular configuration would be (−*s*/2, −*s*/6 × $\sqrt{3}$), (0, *s*/3 × $\sqrt{3}$), and (*s*/2, −*s*/6 × $\sqrt{3}$). Here, *s* represents the separation between the probes. From the fraction of necrotic tissue in the case with a single probe, we can create three identical domains and position the centers of these domains at the positions of the probes in the multi-probe configuration and create a new domain, which is again positioned and has the same dimensions as the starting domain (blue square), whose value for the fraction of necrotic tissue is the sum of the values of all domains composing the new domain.

Full 3D calculations of the electromagnetic field were calculated and available at each time step. The distribution of the electric field vector by volume is sufficient for the complete calculation of the MWA. All effects, including losses and tissue interaction, are included in the volumetric electric field vector distribution. The temperature was calculated for each time step during the MWA, and consequently, the temperature-dependent material parameters changed with time/temperature. For each time step, the electric field was recalculated with new material parameters.

## 3. Results

**Figure 3:**
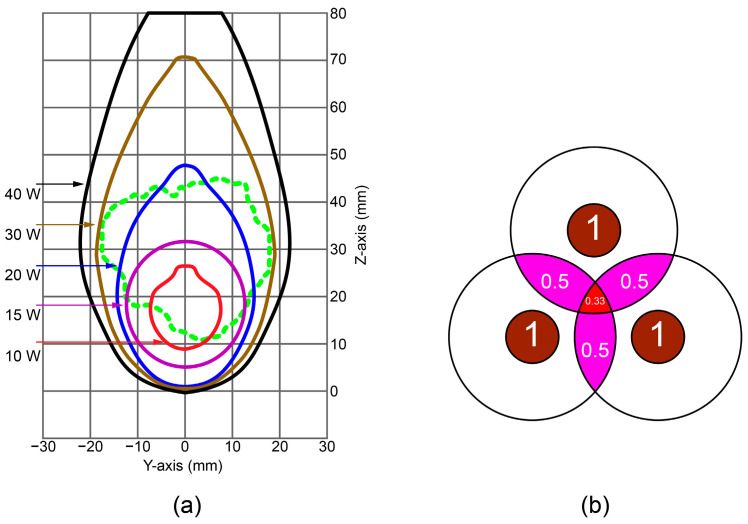
(**a**) Ablation zones created around the tumor (green dashed line) after 600 s of MWA for different input powers. The green dashed curve represents the tumor. (**b**) Diagram representing the overlapped ablation zones from three individual probes with designed zones of different fractions of necrotic tissue.

Figure 3a shows that a 10 W power input resulted in a small, spherical ablation zone with a diameter of approximately 15 mm. An input power of 15 W produced a highly spherical ablation zone with a diameter of approximately 26 mm, thereby increasing the ablation volume by 5.7 times compared with the 10 W case. A 20 W input led to a greater increase in ablation volume, which was 2.23 times larger than that for a 15 W input. However, the growth was more focused on the power source in the longitudinal direction, reaching a length of 46.6 mm, while the maximum diameter in the transverse plane was around 30 mm. An input power of 30 W resulted in an ablation volume that was 2.37 times larger than that of the 20 W case, whereas a 40 W power resulted in a total volume of 1.65 times larger than that of the 30 W case. In 30 W ablation, the tumor was not entirely contained, and small transverse plane parts appeared. On the other hand, for 40 W, the entire tumor can be easily ablated; however, the volume of ablation is 5.3 times larger than the tumor volume; hence, there is a particularly large amount of damaged and healthy tissue. As power increases, the ablation effect tends to extend primarily in the longitudinal direction around the probe, leading to significant damage to the healthy tissue.

Based on the nearly triangular shape of the tumor in the *xy*-plane (Figure 1b), using three probes in a triangular configuration appears to be an optimal choice. Figure 3b shows the ideal circular distribution of the ablation zones originating from the three individual probes, which allowed us to estimate total necrosis, including overlapping areas and combined ablation zones. The probe is in the center of each circle, and the proximity of the probe radiation causes it to have the highest intensity, and ablation will occur rapidly. Small red circles with fractions of tissue damage close to 1 represent these zones. Moving away from this zone, we would have zones with a fraction of tissue damage ranging from 1 to 0. In the multiple-probe configuration, the key element is the overlap of the ablation zones. For example, if two overlapping zones each have a necrotic tissue fraction of approximately 0.5, the combined necrotic tissue fraction would be approximately 1. At the same time, if we have three probes, we would only need to have a fraction of the necrotic tissue of approximately 0.33 in the overlapping zones to reach full tissue necrosis.

**Figure 4:**
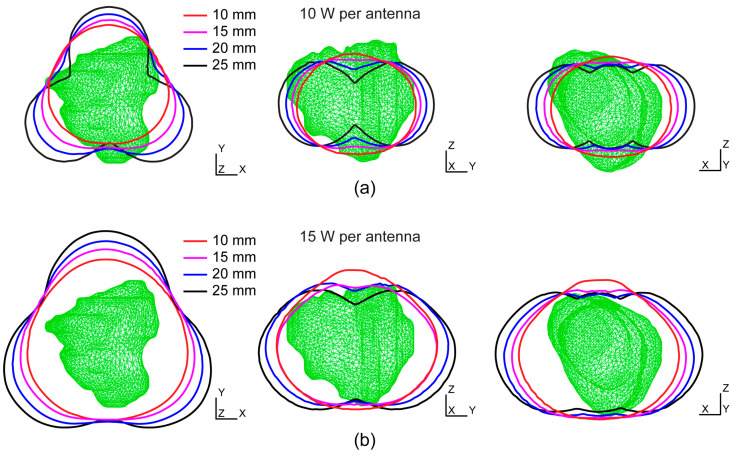
Overlapping ablation zones from three individual probes estimated from single-probe simulations with different separations and input powers of (**a**) 10 W and (**b**) 15 W for each probe. The green triangular surfaces represent tumors from the database [31].

Figure 4a shows the estimated ablation shapes in different planes for 10 W per antenna and separations of 10–25 mm. In all cases, the tumor was larger than the ablation zone. For 10 mm separation, the ablation was highly spherical and unified, and there was no differentiation between the ablation zones originating from the individual probes. With increasing separation, the unity is broken, especially for separations larger than 20 mm, which is undesirable because it can leave large central parts of the tumors untouched. For 15 W per antenna, the entire tumor in the *xy*-plane was within the ablation zone, as shown in Figure 4b. In the *zy*-plane for separation equal to or larger than 20 mm, the created ablation zones are non-unified compared with the large spherical ablation zone created for 10 mm. Based on the analysis of these results, we proceed with the full 3D FEM simulation of the model with three probes, 15 W per antenna, and separations of 10 and 15 mm.

**Figure 5:**
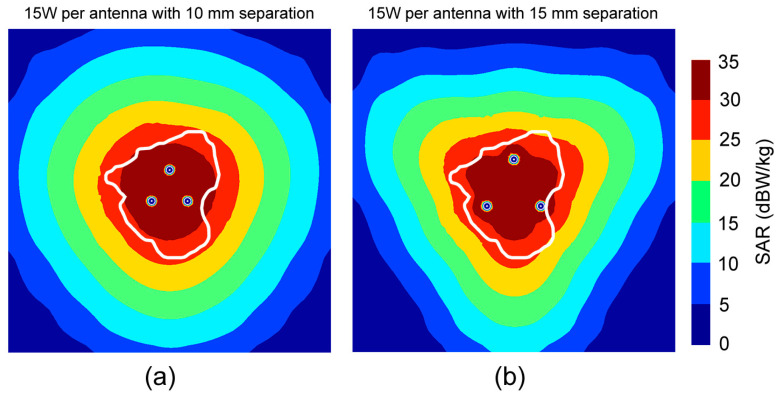
SAR patterns for three probes in a triangular configuration in the *xy*-plane with 15 W per probe and separations of (**a**) 10 mm and (**b**) 15 mm. The white line indicates the tumor from the database [31].

The full 3D FEM simulation results of the SAR for the three simultaneous probes in a triangular configuration with 15 W for each probe and two separations are shown in Figure 5. The SAR represents the interaction of electromagnetic radiation with living tissue and closely follows the temperature and ablation distribution. Tissue within zones with levels of at least 25 dBW/kg are practically guaranteed to be ablated. We can see that with a 10 mm separation, the SAR shape is spherical, while with a 15 mm separation, it is triangular, and there is significant concentration of the high-level SAR in the zones between the probes and at the intersections.

**Figure 6:**
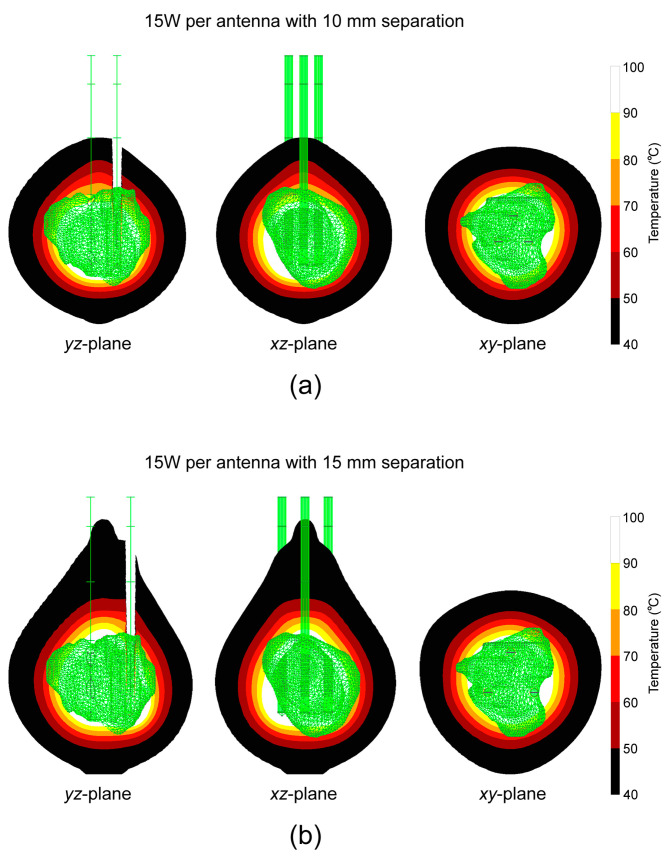
Temperature distribution for 15 W per probe and separation of (**a**) 10 mm and (**b**) 15 mm after 600 s in the *yz*-plane, *xz*-plane, and *xy*-plane. The green triangular surface represents the tumor (from the database [31]).

The absorbed energy converted into thermal energy causes an increase in the tissue temperature. The temperature distribution after 600 s of MWA with an input power of 15 W and the antenna separation of (a) 10 mm and (b) 15 mm is shown in Figure 6. For both separations, the entire tumor was within the region at temperatures higher than 60 °C, which guarantees instantaneous cell death. The highest temperature is near the antenna slots and decreases as the distance from the antenna increases. 

**Figure 7:**
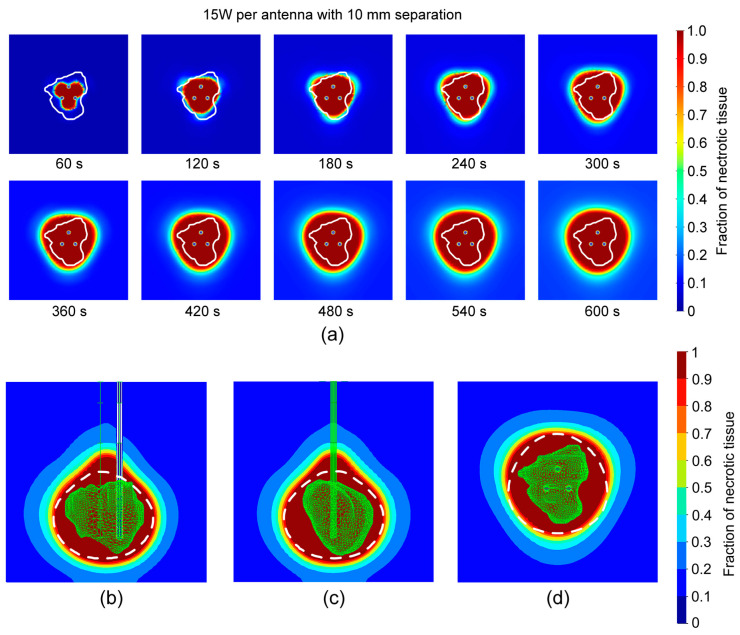
(**a**) Change in the fraction of necrotic tissue with time for 15 W per probe and separation of 10 mm during 600 s in the *xy*-plane. The white line indicates the tumor [31]. A fraction of necrotic tissue distribution after 600 s in the (**b**) *yz*, (**c**) *xz*-plane, and (**d**) *xy*-plane. The white dashed lines show predictions from Figure 4b.

Figure 7a shows that the fraction of tissue necrosis followed the temperature distribution with time, starting with the Y-area between the probes after 60 s, expanding to areas around the probes after 120 s, creating a large triangular formation after 180 s, which changed to a spherical shape later. As can be seen from Figure 7b–d, the entire tumor is inside the ablation zone. The total ablation volume was 34.6 cm^3^.

**Figure 8:**
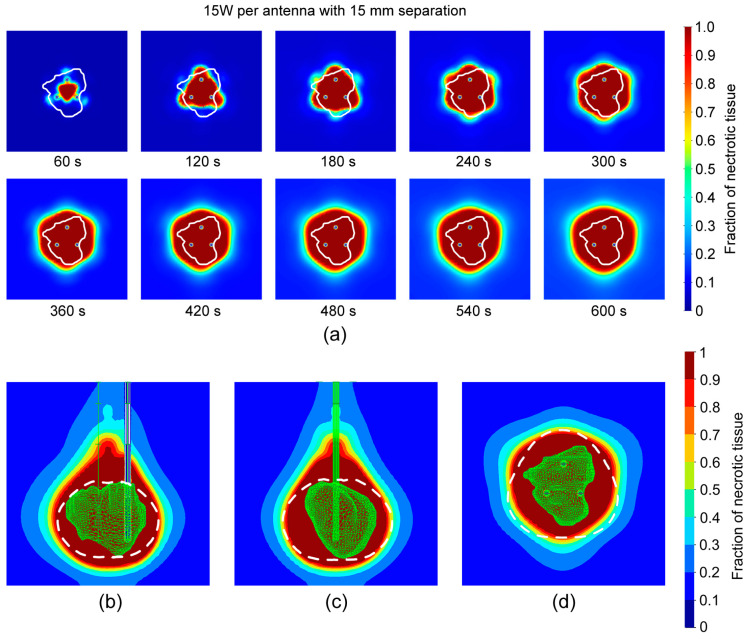
(**a**) Change in the fraction of necrotic tissue with time for 15 W per probe and separation of 15 mm during 600 s in the *xy*-plane. The white line shows the tumor [31]. A fraction of necrotic tissue distribution after 600 s at (**b**) *yz*, (**c**) *xz*-plane, and (**d**) *xy*-plane. The white dashed lines represent predictions from Figure 4b.

Observing tissue necrosis in Figure 8a, we can see that during ablation, the triangle shape between the probes later expands with the area around the probes, forming a hexagonal shape that later expands to a more spherical shape. From Figure 8b–d, we can see that the entire tumor was ablated with a larger ablation size of 38.9 cm,^3^ compared with the case with a 10 mm separation. Figure 7 and Figure 8 show a comparison of the ablation results and the rough estimation based on a single probe (black dashed lines). In both cases, the lateral dimensions were reasonably estimated, whereas the longitudinal size of the ablation was larger in the full FEM simulation, especially in the case with the 15 mm separation.

The parameters that characterize the tissue damage *VD* and *DT* were calculated using Equations (5) and (6). The values for single-antenna, two-antenna, and three-antenna configurations are shown in Table 1. The greatest damage to healthy tissue occurs with a single-antenna configuration. The use of two- and three-antenna configurations causes less damage to the healthy tissue. When three antennas are used, the amount of damaged healthy tissue was less than 2%. As compared with other results, Cazzato and co-workers reported volumetric damage around 237.5% and 337.5% that corresponds to two- and three-antenna liver tumor ablation [10]. For two-antenna ablation, Andresciani et al. found 246% volumetric damage. Manuchehrabadi and Zhu obtained an amount of damaged healthy tissue less than 5% with 100% tumor volume damage [39].

## 4. Conclusions

In this study, we investigated several treatment scenarios for real large elongated tumors (from the database [31]) using three identical parallel-positioned probes with multi-slot coaxial antennas. Three-dimensional simulations with both tumoral and healthy tissue and antenna design were performed using a software framework based on open-source components [26,27]. We assumed that, for large tumors, MWA achieved the best results when multiple probes were used simultaneously to exploit the synergistic effects of the overlapping radiation fields and create an efficient, targeted ablation zone. The 10-slot design helps focus energy on a specific area while minimizing damage to surrounding healthy tissues.

For a fixed duration of the MWA procedure with a single fixed probe, an increase in the input power alone was sufficient to increase the ablation size. Using multiple probes enables the spatial distribution of total power and the ability to customize the ablation shape based on tumor shape and orientation. The main issue when using multiple probes is to find the optimal number of probes, power, and spatial distribution. The number of potential combinations can be quite overwhelming. The accurate shape of the ablation can also be assessed using the numerical FEM approach in the same manner as in the case with a single probe, but with each probe’s numerical complexity increasing considerably; hence, blindly checking multiple combinations is highly inefficient. The number of possible array configuration of different spatial and power distributions is extremely large. We used the SAR results obtained from the single probe to estimate the optimal array configuration and confirmed the validation of the approach by running a full 3D simulation with selected configurations.

Moving away from the radiation source, the fraction of tissue damage decreases; however, in a multi-probe setup, the overlap of lower-damage zones can still cause complete tissue necrosis. For example, if n overlapping zones each have a fraction of tissue damage of 1/*n*, the sum of the fractions will be 1, indicating complete tissue ablation. In summary, the results indicate that higher-power inputs (30 W and 40 W) are more likely to provide complete tumor coverage than lower-power inputs (10 W, 15 W, and 20 W).

In a multi-probe setup, each probe generates a separate ablation zone. The goal is to unite these individual ablation zones into a single, large, unified, and uninterrupted ablation zone that covers the entire tumor. Therefore, the individual ablation zones created by each probe should be near each other. The combined ablation zone can be significantly larger than the simple sum of the volumes obtained from the individual probes. These results can be attributed to the synergistic effects of the combined radiation from multiple probes. Our results showed that volumetric damage and the amount of damaged healthy tissue were greatest with microwave ablation using a single antenna. The damage to healthy tissue decreases for two and three antennas. For the three-antenna ablation, the amount of damaged healthy tissue was less than 2%. Presented results indicate that triple-antenna MWA provides complete ablation of a large tumor with a small risk of unnecessary damage and predictable ablation zones.

## Figures and Tables

**Table 1:** *VD* and *DT* after MWA performed using single-antenna, two-antenna, and three-antenna configurations.

Configuration	*VD*	*DT*
Single antenna (40 W)	452.3%	3.4%
Two antennas (15 mm separation and 20 W per probe)	213.1%	1.6%
Three antennas (15 mm separation and 15 W per probe)	194.6%	1.4%
Three antennas (10 mm separation and 15 W per probe)	161.6%	1.2%

## Data Availability

The data are available from the corresponding authors upon reasonable request.
